# The psychosocial situation of families caring for children with rare diseases during the COVID-19 pandemic: results of a cross-sectional online survey

**DOI:** 10.1186/s13023-022-02595-0

**Published:** 2022-12-26

**Authors:** Lydia Rihm, Mareike Dreier, Farhad Rezvani, Silke Wiegand-Grefe, Jörg Dirmaier

**Affiliations:** 1 Department of Medical Psychology, Center for Psychosocial Medicine, University Medical Center Hamburg-Eppendorf, Hamburg, Germany; 2 Department of Child and Adolescent Psychiatry, Psychosomatics and Psychotherapy, University Medical Center Hamburg-Eppendorf, Hamburg, Germany

**Keywords:** Rare disease, COVID-19 pandemic, Family caregiver, Distress, Mental health, Health-related quality of life, Support offerings, Psychosocial care situation

## Abstract

**Background:**

The COVID-19 pandemic is affecting many areas of life and has posed additional strains on the highly vulnerable group of caregivers of children with rare diseases (RDs). The psychosocial situation of the family caregivers deserves more attention, both in research and practice. The current study explores the distress level of caregivers of children with RDs, their psychosocial information needs, and caregiver-reported health-related quality of life (HRQoL) of children with RDs in times of the COVID-19 pandemic.

**Methods:**

Data from a cross-sectional online survey conducted within the German CARE-FAM-NET project (*children affected by rare diseases and their families-network*) between March and August 2020 were examined. The study sample included 149 family caregivers, mostly mothers (83.2%) of 167 children with RDs. The survey assessed demographic and disease-related characteristics, distress and everyday problems of caregivers (Distress Thermometer for Parents; scale 0–10), psychosocial information needs (self-developed items; scale 0–100), and caregiver-reported HRQoL of the children with RDs (DISABKIDS Chronic Generic Measure, short-form; scale 0–100). Using descriptive statistics, we analyzed the psychosocial situation of families during the COVID-19 pandemic. We further conducted correlation analysis to investigate interrelations.

**Results:**

The distress level among caregivers was high (*M* = 6.84, *SD* = 2.43); 89.6% reported clinical distress (≥ 4). Everyday problems (e.g., sleep problems, fatigue, being out of shape, fears, feeling tense or nervous, and worry) were frequent. Caregivers reported a wide range of psychosocial information needs. In about half of the children (49.5%), caregiver-reported HRQoL was low, while average HRQoL (*M* = 58.7, *SD* = 19.5) was comparable to parent-reported norm data of children with severe clinical conditions. Distress correlated positively with psychosocial information needs (*r* = 0.40), and negatively with the caregiver-reported HRQoL of the children (*r* =  − 0.46).

**Conclusions:**

This study indicates a high psychosocial burden on family caregivers of children with RDs during the early COVID-19 pandemic, characterized by high distress levels and wide-ranging everyday problems, unmet psychosocial information needs, and reduced caregiver-reported HRQoL in children with RDs. The findings highlight the ongoing need for target group-specific, low-threshold support services (e.g., websites) during and after the pandemic.

**Supplementary Information:**

The online version contains supplementary material available at 10.1186/s13023-022-02595-0.

## Background

Living with a child with a rare disease (RD) represents an immense psychosocial burden for the whole family [1, 2]. Issues specific to RDs include delayed diagnosis, complexity of symptoms, limited medical expertise and barriers in accessing the care system, geographically dispersed patient groups, and ignorance of the general population [3–11]. Parents of children with rare genetic syndromes experience higher levels of distress and mental health difficulties compared to parents of children with other disabilities [12]. Families with children with RDs from nations with well-resourced health care systems, such as Germany [13], the Netherlands [14], and Northern Ireland [15], consistently report not receiving adequate psychosocial care following a diagnosis of an RD. More information on psychosocial support options and social-legal counseling is needed in this population [1, 8, 13, 16–18]. A relationship between access to psychosocial information and distress levels is conceivable. For instance, improved access to information predicted the well-being of parents of children with various disabilities [19]. In a setting of family-centered care, being provided with general information (e.g., on community services) strongly correlated with family empowerment [20]. However, the association between access to psychosocial information and distress has not yet been studied specifically for the unique group of caregivers of children with RDs.

The COVID-19 pandemic and its associated stressors placed a burden on families and negatively affected the mental health of children in the general population [21, 22]. Several studies indicate that COVID-19 restrictions placed an even greater burden on families that were already heavily strained before the pandemic, primarily due to the higher risk of severe illness, disruption of routine care, concerns about safely accessing health care, as well as social and economic hardship [23–26]; for instance, overwhelmed hospitals and delayed examinations affect those most in need of regular medical care [27]. In addition, many of the few existing face-to-face support offerings were canceled due to COVID-19 restrictions. Initial findings indicate that parents of children with a congenital RD had significantly lower quality of life and higher mental health impairment during the first years of the COVID-19 pandemic compared with a control group and norm data [28]. Thus, the COVID-19 pandemic may have further exacerbated health disparities [29]. As the pandemic has simultaneously strained existing healthcare, policy, and research capacity, there is now concern that psychosocial care for families with children with RDs will be further compromised [28].

Pre-pandemic results on the variables of distress and psychosocial information needs among caregivers of children with RDs and HRQoL among their children with RDs are limited, and research on the psychosocial burden during the pandemic is still in its infancy. A more comprehensive understanding of the situation of families living with an RD in times of the COVID-19 pandemic is critical and may highlight the importance of family-centered mental health care. Ameliorating distress in family caregivers could indirectly also benefit the well-being of the children and their adjustment to the disease [30–33] and the well-being of healthy siblings [34].

The current study addresses the distress level and psychosocial information needs among caregivers of children with RDs, and the caregiver-reported HRQoL among their children during the first year of the COVID-19 pandemic. We investigate 1) the level of distress, prevalence, and everyday problems associated with clinical distress among family caregivers of children with RDs; 2) the content and amount of psychosocial information required by caregivers of children with RDs; 3) caregiver-reported HRQoL of children with RDs compared with norm values of children with chronic conditions. We further explore the interrelations between the studied variables. The goal of this work is to inform next steps in developing psychosocial information and support for families of children with RDs during and after the pandemic.

## Methods

### Study design and sample

The current analyses were run on a data set that we collected within the German CARE-FAM-NET project–*Children affected by rare diseases and their families-network* [35]. Ethical approval for all studies linked to the CARE-FAM-NET project was gained from the Ethics Committee of the Medical Chamber Hamburg (PV 5749). All participants provided consent to this study.

A cross-sectional online survey was administered via LimeSurvey (Version 2.62.2+170203) between March and August 2020. The survey included the first COVID-19 wave in Germany (weeks 10–20 of 2020), as well as an interim phase over the summer with fewer cases (weeks 21–39 of 2020) [36]. COVID-19 restrictions during the study period included: school and daycare closures; widespread closures including restaurants and retail, as well as zoos and playgrounds; contact restrictions (max. 2 people or own household)–hence the cancellation of events including leisure and sports activities; wearing mouth and nose protection [36]. We planned the study before the pandemic, which means that no direct questions related to COVID-19 were asked. Participants were recruited via ACHSE–*Alliance of chronic rare diseases*–, the German umbrella organization of support groups concerned with RDs, as well as via additional support groups, social media, and stakeholders in the field of RDs (e.g., associations, practitioners). Relatives that affirmed to care for at least one child (no age restriction) with a diagnosed or probable RD were allowed to participate. Having a confirmed RD diagnosis was not an inclusion criterion, as reasonable suspicion of an RD without being able to give a formal diagnosis is common in the realm of RDs. Due to the nature of the online survey, internet access and sufficient German skills were needed to participate. There were no further in- or exclusion criteria. In total, 202 participants gave informed consent and accessed the online survey. Of these, 24 were excluded from participation because they did not confirm to be a relative caring for a child with an RD. In addition, 29 dropped out before answering any of the main variables of interest and were excluded from the following analyses. Of the remaining 149 participants, 133 completed the survey in full; 16 participants completed only parts of the survey, which we included in our analyses.

### Measures

The online survey incorporated validated instruments as well as self-developed items derived from a qualitative pre-analysis [see Additional file 1]. Due to adaptive questioning (e.g., date of diagnosis was only asked when previously indicated that a diagnosis had been confirmed; the questionnaire on the HRQoL of children was only administered to parents with children aged 4 years and older), the number of items varied from 107 to 122 questions. Furthermore, some items had to be answered for each child with RD in the family. The average completion time was approximately 20 min.

#### Sociodemographic and disease-related characteristics

Sociodemographic data included sociodemographic background information; confirmed diagnosis (*yes*/*no*); time since diagnosis (if a diagnosis was confirmed); in- and outpatient treatment of the child(ren) with RD in the last two weeks/ the last year (*yes*/*no*); and physical, mental, and social functional impairment of the child(ren) with RD (*yes/no/no comment*). Information on the diagnosis of the child was not collected, as the anonymity of the participants would then no longer have been guaranteed due to the rarity of some RDs in Germany.

#### Distress of caregivers

An adapted version of the Distress Thermometer for Parents (DT-P, [37]) was used to assess the distress level of family caregivers. The DT-P is a well-validated, brief screening instrument identifying overall distress and its sources in parents of children with chronic diseases and has shown acceptable psychometric properties [38]. It was originally adapted from the Distress Thermometer frequently used in oncology medical care [39, 40] and has already been used in samples of parents of children with diverse RDs [30, 41].

The DT-P consists of a thermometer and a problem list. On the thermometer, caregivers rate their overall distress (physically, emotionally, socially, and practically in general) experienced in the past week on a visual analog scale ranging from *no distress* (0) to *extreme distress* (10), with a score ≥ 4 indicating clinically elevated distress. The cutoff was determined in the original study with parents of children with chronic diseases [37] and has also been used in samples of parents of children with RDs [30, 41]. The problem list inquires about the experience of everyday problems in the domains *practical*, *family/social*, *emotional*, *physical*, *cognitive*, and *parenting* problems (separate for < 2-year-old and ≥ 2-year-old children) over the past week (*yes* = 1, *no* = 0), e.g., “feeling tense or nervous”. A total problem score (sum of reported problems) and problem domain scores (sum of reported problems per domain) can be calculated. Correlations between the distress score and problem list scores provide preliminary evidence as to whether experiencing problems from the list might be related to elevated distress levels and which domains might be particularly relevant. In addition, item scores (percentage of *yes* answers for individual items) provide important insight into which problems are most prevalent.

For the present survey, the project team translated the original English wordings into a German version. The translation process was monitored by the principal investigator. In the case of ambiguous translations (e.g., “out of shape/condition”), we discussed options and decided jointly. We added three items to the *emotional* problem domain from the Distress Thermometer for Caregivers [42]: grief, worry, and stigma. For the *parenting* problem domain, we used the items of the older children and supplemented them with two items from the younger children (the sleeping of the child and feeling connected to the child). The additional questions section was removed.

In the current study, reliability analyses showed very good internal consistency for the total problem score (39 items, Cronbach’s α = 0.91). The problem domains *physical* (7 items), *cognitive* (2 items), and *emotional* problems (12 items) reached satisfactory to good internal consistency (Cronbach’s α = 0.72, 0.72, 0.82, respectively), while the domains *practical* (7 items), *social/family* (4 items) and *parenting* problems (7 items) reached acceptable Cronbach’s α values of 0.58, 0.61, 0.65, respectively.

#### Psychosocial information needs of caregivers

Caregivers were asked about their present information needs regarding psychosocial issues that we compiled based on a qualitative pre-analysis of semi-structured telephone interviews with 10 relatives of children with RDs and eight experts in the field [for details see Additional file 1]. We used a total of 43 items to measure information needs in the following domains: *navigating the health care system* (17 items), *psychosocial strain in the family* (11 items), *strengthen yourself to be strong for others* (8 items) and *further support offerings* (7 items). One example item is: “Information about how I can cope with my feelings after the diagnosis”. See [Additional file 2] for the original German version and [Additional file 3] for a list of all items. The response format was a five-point Likert scale with options ranging from *no information needed* (1) to *in-depth information needed* (5). For each information domain and the overall information need, a transformed sum score ranging from 0 to 100 can be calculated from the raw sum scores, with higher values indicating a higher information need. Average ratings for individual items represent item scores.

In the current study, reliability analyses showed very good internal consistency for the overall psychosocial information need scale (43 items, Cronbach’s α = 0.95) as well as good internal consistencies for the subscales on the domain level, with Cronbach’s α = 0.90; 0.89; 0.90; 0.84 for the respective subscales as listed above.

To assess the provision of psychoeducational internet offerings for the caregivers, they were additionally asked whether they knew of helpful web pages on psychosocial burdens (*yes*/*no*).

#### Health-related quality of life of children with RDs

The HRQoL of children was assessed by the German proxy-report short-form of the DISABKIDS Chronic Generic Measure–the DCGM-12-p [43–45]. The DISABKIDS Group Europe specifically developed the DISABKIDS measures to assess the HRQoL of children and adolescents with chronic conditions. The measures have shown good psychometric properties [43, 44]. The DCGM-12 consists of 10–12 Likert-scaled items evenly assigned to *mental* (independence; emotion), *social* (inclusion; exclusion), and *physical* domains (limitation; treatment–only if medication intake was affirmed beforehand) relevant to HRQoL (e.g., “Is your child unhappy because of their illness”). The frequency of the behaviors or feelings asked about is rated from *never* (1), *rarely* (2), *quite often* (3), *very often* (4) to *always* (5), based on the last four weeks. The DCGM-12 holds a one-dimensional factor structure so that a transformed composite score from all items (range 0–100) represents the overall caregiver-reported HRQoL of the children, with higher scores indicating higher HRQoL. Based on a recommendation by Muehlan [44], we additionally report a second total score consisting of the first 10 items (without medication; DCGM-10). As comprehensibility for children was not a necessity, we administered the DCGM-12-p to all caregivers of children older than 4, rather than using the smiley version for caregivers of children between 4 and 7 years, to ensure the comparability of the data. We used the DISABKIDS field study sample (children and adolescents with a chronic condition, parent-proxy report) as a reference [43, 44]. A general cut-off score to differentiate low from high HRQoL does not exist. Instead, low caregiver-reported HRQoL was determined by an individual T-score < 40 (1 *SD* below the mean) on the standard deviation scale of the DISABKIDS field study sample [43]. In the current study, reliability analyses showed good internal consistency for the DCGM-12 score (Cronbach’s α = 0.86) as well as the DCGM-10 score (Cronbach’s α = 0.90).

### Statistical analyses

Statistical analyses were carried out with SPSS 27 (IBM Corp., Armonk, NY). We used descriptive statistics to depict the psychosocial situation of the families (demographic and disease-related characteristics; distress level, everyday problems, and psychosocial information needs of the caregivers; caregiver-reported HRQoL of the children), reported as means and standard deviations for continuous variables and counts and percentages for categorical variables. In the case of non-normality of continuous data, the median and interquartile range were additionally calculated, while the mean was retained to allow for comparison with previously published data.

We compared the caregiver-reported HRQoL of the children to the parent-reported HRQoL norm data of children and adolescents (8–16 years old) from several European countries with mild, moderate, or severe chronic health conditions in the DISABKIDS field study sample [43]. Differences in mean scores were analyzed with multiple unpaired t-tests; or Welch tests in case of heterogeneous variances by using the indicated summary statistics. Due to the explorative nature of analyses, no correction for the cumulation of alpha error was performed.

We examined the intercorrelations between the study variables using the appropriate correlation coefficients (Pearson’s correlation for metric variables, point-biserial/biserial correlation for metric and (artificial) binary variables; Kendall’s Tau rank correlation for metric and ordinary variables; Phi for binary variables; rank-biserial correlation estimated with Spearman’s correlation rho for ordinal and binary data).

For all analyses, *p* values < 0.05 (two-tailed tests) were considered statistically significant.

## Results

### Sociodemographic and disease-related characteristics

Sociodemographic data and disease-related characteristics are presented in Table 1. Of the 149 participants included, 65 (43.6%) completed the questionnaire during the first COVID-19 wave in Germany (weeks 10–20 of 2020), and 84 (56.4%) during the following interim phase over the summer with fewer cases (weeks 21–39 of 2020). Data of 16 participants were missing for part of the demographic variables, leading to a derogated *N* = 133, whenever noted. Participants were mostly mothers (83.2%) caring for a total of 167 children with a confirmed (89.8%) or probable (10.2%) diagnosis of an RD. The mean age of participants was 43.1 years (*SD* = 8.83, *N* = 133), ranging from 16 to 67 years. All participants were living in Germany (*N* = 133), most were married or living in partnership (84.2%, *N* = 133), and their level of education was predominantly high (48.1% university degree or higher, *N* = 133).

**Table 1:** Sociodemographic and disease-related characteristics of the study sample

Characteristics	*n*	%
*Relation of caregiver to the child with RD*		
Mother	124	83.2
Father	21	14.1
Foster mother	1	0.7
Grandmother	1	0.7
Brother	2	1.3
*Marital status of caregiver *^*a*^		
Single/divorced/widowed	21	15.8
Married/partnered	112	84.2
*Highest educational level of caregiver *^*a*^		
Basic school qualification (usually 9 years of High School)	5	3.8
Intermediate school qualification (10 years of High School)	28	21.1
Higher education entrance qualification (12–13 years of High School)	36	27.1
University degree or higher	64	48.1
*Treatment of at least one of the children with RD per family *^*b*^		
Outpatient treatment in the last year	133	89.3
Outpatient treatment in the last two weeks	49	32.9
Inpatient treatment in the last year	82	55.0
Inpatient treatment in the last two weeks	8	5.4
*Functional impairments of the children with RD *^*c*^		
Physical/ motor function only	20	12.0
Mental development only	2	1.20
Social functioning only	8	4.80
Physical/ motor function plus mental development	8	4.80
Physical/ motor function plus social functioning	19	11.4
Mental development plus social functioning	13	7.80
Impairment in all three domains	59	35.3

*RD* Rare disease. *N* = 149. Caregivers were on average 43.1 years old (*SD* = 8.83, *N* = 133)

^a^ derogated *N* = 133. ^b ^Reflects the number and percentage of participants answering “yes”. ^c ^*N* = 167 children with RDs that were on average 10.4 years old (*SD* = 7.99); reflects the number and percentage of respective nested “yes” answers

On average, there were 1.12 children (*SD* = 0.38, *Mdn* = 1, *IQR* = 1–1) with an RD per family. In 59.7% of the families, at least one sibling without RD was living in the household. The mean age of the children with RDs was 10.4 years (*SD* = 8.00, *Mdn* = 9, *IQR* = 4–16, *N* = 167), ranging from 0 to 47 years with a vast majority being up to 21 years old (92.2%). In the 136 families that already received a diagnosis, the average time since diagnosis was 7.11 years (*SD* = 6.93, *Mdn* = 4.71, *IQR* = 2.17–11.04), ranging from 0.34to 46.7 years. On average, the (oldest) child in the family with an RD was 3.99 years old (*SD* = 5.41, *Mdn* = 2.75, *IQR* = 0–6) when the family first received an RD diagnosis. A vast majority of the children (91.3%) were in (in- or outpatient) treatment within the last year. Of the 167 children, 129 (77.2%) suffered from functional impairment in at least one of the queried domains physical/motor function (63.5%), mental development (49.1%), social functioning (59.3%). In total, 18.0% were impaired in only one domain, 24.0% in two domains, and 35.3% in all three domains.

### Distress of caregivers

The descriptive statistics of the DT-P (thermometer score, problem domain scores) are presented in Table 2. Clinical distress (thermometer score ≥ 4) was reported by 89.6% of the caregivers.

**Table 2:** Overall distress and everyday problems experienced by family caregivers of children with RDs

	M (SD)	Possible range	Observed range
Distress thermometer score (overall distress) ^a^	6.84 (2.43)	0–10	0–10
*Mdn* (*IQR*) ^a^	8 (5.75–8)		
Total problem score	17.4 (8.51)	0–39	0–36
Practical problems	1.82 (1.30)	0–7	0–5
Physical problems	3.88 (1.97)	0–7	0–7
Family/social problems	1.54 (1.30)	0–4	0–4
Cognitive problems	0.99 (0.89)	0–2	0–2
Emotional problems	5.40 (3.16)	0–12	0–11
Parenting problems	2.60 (1.84)	0–7	0–7

*RD* Rare disease. *N* = 136. Distress and problem scores were measured with the Distress Thermometer for parents (DT-P). For the thermometer score, higher scores = more distress. Problem domain scores represent the average number of problems in the respective problem list domain that caregivers experienced in the last week including today (*yes*-answers per participant). Please note that mean problem domain scores are dependent on the respective scale length

^a ^Reduced *N* = 134; median (*Mdn*) and interquartile range (*IQR*) are reported because the distribution of thermometer scores was left-skewed (skewness =  − 1.06, *SE* = 0.21)

**Fig. 1:**
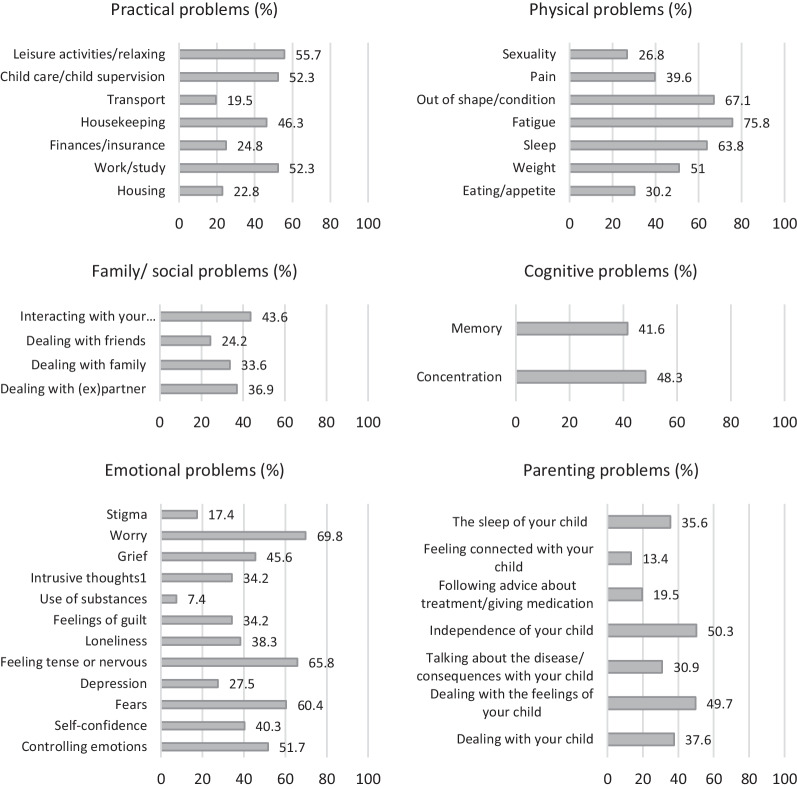
Everyday problems experienced by family caregivers of children with RDs. *Note* N = 136. Items are from the problem list of the Distress Thermometer for Parents (DT-P). Percentages represent the proportion of caregivers that experienced the respective problem in the last week including today (i.e., yes-answers per item)

The thermometer score was strongly related to the total problem score (*r* = 0.68) and to the domain scores of emotional (*r* = 0.61) and parenting problems (*r* = 0.52). The domain scores of practical (*r* = 0.43), physical (*r* = 0.48), social/family (*r* = 0.47), and cognitive problems (*r* = 0.42) were moderately correlated to the thermometer score. All correlations were statistically significant (*p’*s < 0.001). Item scores (percentage of *yes*-answers for individual items) represent the proportion of caregivers who experienced each problem in the last week (including today), presented in Fig. 1. The most frequent problems (> 60%) in the current sample were sleep problems, fatigue, being out of shape, fears, feeling tense or nervous, and worry.

### Psychosocial information needs of caregivers

**Fig. 2:**
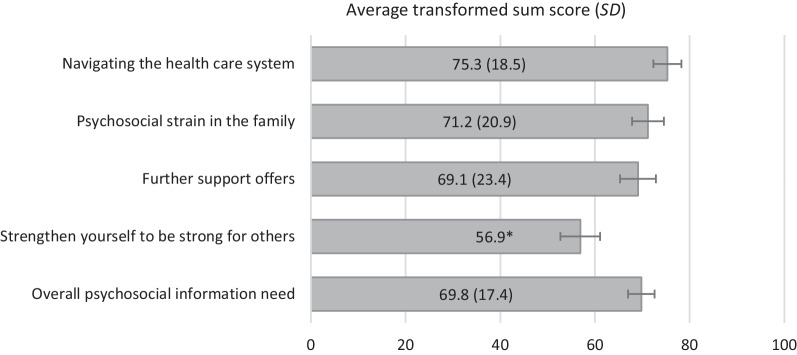
Overall and domain-specific psychosocial information needs of family caregivers of children with RDs. *Note* RD: rare disease. N = 149. Psychosocial information needs were measured by 43 five-point Likert scaled items distributed over the four displayed domains (17;11;7;8 items respectively from top to bottom) with answer options ranging from 1 (no information needed) to 5 (in-depth information needed). Scores were combined to transformed sum scores each ranging from 0 to 100, with higher scores indicating higher information need. Error bars indicate 95% confidence intervals. *SD = 26.1

Figure 2 presents the average psychosocial information need scores of the caregivers (overall and domain-specific transformed sum scores, range 0–100). Information needs were highest for the domain *navigating the health care system*, followed by the domains *psychosocial strain in the family, further support offerings,* and *strengthen yourself to be strong for others*.

Descriptive statistics on item level are presented in Table 3 for the items with the highest ratings per domain. A complete list of all items including descriptive statistics can be found in [Additional file 3].

**Table 3:** Psychosocial information needs of family caregivers of children with RDs on item level

	M (SD)	n (%) 4/5^a^	Mdn (IQR)
*Navigating the health care system*			
Information about benefits within the framework of the “federal participation act” [Bundesteilhabegesetz]	4.48 (1.00)	125 (83.9)	5 (4–5)
Information about special rehabilitation measures (e.g., parent–child measures, rehabilitation measures specifically for children with disabilities)	4.49 (0.91)	131 (87.9)	5 (4–5)
Information about support options without a confirmed diagnosis (e.g., contact points for children with unclear diagnoses)	3.23 (1.61) 4.77 (0.60) ^b^	73 (49.0) 12 (92.3) ^b^	3 (1–5) 5 (5–5) ^b^
*Psychosocial (i.e., mental, emotional, social) strain in the family*			
Information about how I can cope with emotional strains (e.g., fear, sadness, anger, loneliness) in connection with the disease of the child	4.11 (1.04)	114 (76.5)	4 (4–5)
Information about possible emotional reactions of siblings due to the disease of the child (e.g., fear, sadness, anger, loneliness)	3.90 (1.44) 4.45 (0.94) ^c^	106 (71.1) 77 (86.5) ^c^	5 (3–5) 5 (4–5) ^c^
Information about how to support the social inclusion of the child	4.07 (1.08)	106 (71.1)	4 (3–5)
*Strengthen yourself to be strong for others*			
Information about how I can prevent mental illness	3.42 (1.31)	76 (51.0)	4 (2–5)
Information about how I can strengthen siblings	3.67 (1.50) 4.25 (1.00) ^c^	100 (67.1) 77 (86.5) ^c^	4 (3–5) 4 (4–5) ^c^
Information about how I can combine work and caring for the child	3.57 (1.48)	87 (58.4)	4 (2.5–5)
*Further support offerings*			
Information about support options in everyday life	4.07 (1.16)	110 (73.8)	4 (3–5)
Information about regular care options for the child	3.83 (1.33)	96 (64.4)	4 (3–5)
Information about local points of contact	3.85 (1.32)	96 (64.4)	4 (3–5)

*RD* rare disease. *N* = 149. The table shows a selection (based on the highest ratings per domain) of the 43 items used to assess psychosocial information needs. Items were distributed over the four displayed domains (17;11;8;7 items respectively from top to bottom) with answer options ranging from 1 (*no information needed*) to 5 (*in-depth information needed*). A complete list of the items can be found in [Additional file 3]. The median and interquartile ranges are reported in addition to average ratings, due to the non-normality of ratings. Some item scores are additionally reported for sub-samples, as the content assumedly only impacts these defined subgroups

^a^ number (percentage) of ‘4’ or ‘5’ ratings for the respective item. ^b ^only caregivers with children without a confirmed diagnosis, *N* = 13. ^c ^only caregivers with healthy siblings in the household, *N* = 89

Overall, 34.9% of the items had mean scores above 4; 44.2% between 3.50 and 4; 16.3% between 3 and 3.49, and only two of the 43 items (4.7%) reached mean scores below 3.

Four participants out of 140 (2.9%) affirmed knowing helpful webpages on the subject of psychosocial burdens, whereas 136 (97.1%) negated.

### Health-related quality of life of children with RDs

In total, 107 caregivers completed the short version of the DISABKIDS chronic generic measure (DCGM-12 proxy) for their oldest child with RD above the age of four years. Of those, 71 completed the two questions on medication. The mean age of the respective children was 12.7 years (*SD* = 7.71, *Mdn* = 10.0, *IQR* = 6–17), ranging from 4 to 47 years. Average caregiver-reported HRQoL scores in the current sample were *M* = 58.7 (*SD* = 19.5, *N* = 107) for the DCGM-12 score (i.e., including medication items if applicable) and *M* = 57.6 (*SD* = 21.4, *N* = 107) for the DCGM-10 score (i.e., excluding medication items for all participants). Children without medication reached higher caregiver-reported HRQoL scores than children with medication (*M* = 70.3, *SD* = 16.4, *N* = 36 vs. *M* = 52.8, *SD* = 18.3, *N* = 71), *t*(105) = 4.85, *p* < 0.001, *d* = 0.99.

The average caregiver-reported HRQoL score of the current sample was lower than that of the total DISABKIDS field study sample [43] (*M* = 74.6, *SD* = 17.0, *N* = 660, *p* < 0.001, *d* = 0.92), and that of the DISABKIDS field study sub-samples including children and adolescents with moderate conditions (*M* = 73.8, *SD* = 15.9, *N* = 260, *p* < 0.001, *d* = 0.89), and mild conditions (*M* = 77.9, *SD* = 16.3, *N* = 334, *p* < 0.001, *d* = 1.12), whereas the score was comparable to that of the DISABKIDS field study sub-sample of children and adolescents with severe clinical conditions (*M* = 61.4, *SD* = 18.0, *N* = 66, *p* = 0.369, *d* = 0.14). The T-scores in the current sample, determined by the standard deviation scale of the reference sample [43], ranged from 25.0 to 63.8 (only children without medication) and 22.1–60.5 (only children with medication). Overall, 49.5% of the children had a T-score < 40 (i.e., more than one SD below the respective reference) which was considered as low HRQoL (19.4% of the children without medication and 64.8% of the children with medication).

### Exploratory analysis of the associations between the distress of caregivers, the health-related quality of life of children with RDs, and the information needs of caregivers

An intercorrelation matrix including all study variables is presented in Table 4. The distress level of the caregivers was moderately to strongly associated with their overall psychosocial information need (*r* = 0.40, *p* < 0.001), the caregiver-reported HRQoL of their children with RDs (*r* =  − 0.46, *p* < 0.001), and physical- (*r*_b_ = 0.30, *p* = 0.007), mental- (*r*_b_ = 0.35, *p* = 0.001) and social impairments (*r*_b_ = 0.52, *p* < 0.001) of their children.

**Table 4:** Intercorrelations between demographic, caregiver and child factors

Variable	*n*	1	2	3	4	5	6	7	8	9	10	11	12	13
1. Distress (cg)	134	–												
2. PSIN (cg)	149	0.40^***^	–											
3. HRQoL (c) Impairments (c)	107	− 0.46^***^	0.18	–										
4. Physical function ^a^	149	0.30^**^	0.32^**^	− 0.55^***^	–									
5. Mental function ^a^	149	0.35^**^	0.19	− 0.37^**^	0.36^***^	–								
6. Social function ^a^	149	0.52^***^	0.25^*^	− 0.68^***^	0.38^***^	0.54^***^	–							
7. Mother vs father ^b^	145	− 0.16	− 0.11	0.19^*^	− 0.16	− 0.07	− 0.09	–						
8. Hospitalization (past year, c) ^a^	149	0.18	0.22^*^	− 0.31^*^	0.29^***^	0.03	0.13	0.02	–					
9. Age (cg)	133	− 0.03	− 0.05	− 0.03	− 0.20	− 0.02	0.02	0.17^*^	− 0.31^**^	–				
10. Time since diagnosis	136	− 0.04	− 0.13	− 0.04	− 0.07	0.26^*^	0.18	0.09	− 0.30^**^	0.56^***^	–			
11. Age (c) ^c^	149	0.02	− 0.05	− 0 .16	− 0.13	0.04	0.11	0.04	− 0.31^**^	0.75^***^	0.75^***^	–		
12. Children per family ^d^	149	0.13	0.03	− 0.01	0.03	0.08	0.22^*^	− 0.06	0.09	0.01	0.00	− 0.04	–	
13. Education level (cg) ^e^	133	− 0.05	0.08	− 0.08	− 0.01	− 0.01	− 0.01	0.09	0.03	− 0.07	− 0.11	− 0.19^**^	− 0.09	–
14. Partnership (cg) ^a^	133	0.06	0.04	− 0.13	0.11	− 0.09	− 0.09	− 0.02	0.17^*^	− 0.23^**^	− 0.27^**^	− 0.28^**^	0.19^*^	0.15

Correlation coefficients: *r*, Pearson’s correlation coefficient for metric variables; *r*_b_, biserial correlation coefficient for metric and artificial binary variables; *r*_pb_, point-biserial correlation coefficient for metric and binary variables; *r*_τ_, Kendall’s Tau rank correlation coefficient for metric and ordinary variables; *r*_ϕ_, Phi-coefficient for binary variables; *r*_rb_, rank-biserial correlation coefficient estimated with Spearman’s correlation coefficient rho for ordinal and binary data

Distress = Thermometer score (0–10) on the DT-P, Distress Thermometer for Parents

Abbreviations: (cg), caregiver. (c), children with rare disease. PSIN, overall psychosocial information need (transformed sum score). HRQoL, caregiver-reported Health-related quality of life measured with the DISABKIDS chronic generic measure, short version-proxy (DCGM-12)

^a ^0 = *no*, 1 = *yes*. ^b^0 = *mother*, 1 = *father*. ^c ^Mean age of children with RD per family in case of more than one child with RD. ^d^ Total number including healthy children

^e ^1 = *basic school qualification*, 2 = *intermediate school qualification*, 3 = *higher education entrance qualification*, 4 = *university degree or higher*

^***^p < 0.001 (two-tailed). ^**^p < 0.01 (two-tailed). ^*^p < 0.05 (two-tailed). Significant correlations (*p* < 0.05) are presented in bold

Further, psychosocial information needs were moderately associated with social and physical impairments of the children, while there was a small, however non-significant, association with mental impairment of the children (physical: *r*_b_ = 0.32, *p* = 0.003; mental: *r*_b_ = 0.19, *p* = 0.061; social: *r*_b_ = 0.25, *p* = 0.018). Moreover, there was a small to moderate correlation between psychosocial information needs and the caregiver-reported HRQoL of children with RD, although not statistically significant (*r* = − 0.18, *p* = 0.069).

## Discussion

The psychosocial situation of family caregivers of children with RDs is severely strained, even more so in times of the COVID-19 pandemic. The current study found high distress levels among family caregivers, their manifold psychosocial information needs, and impaired caregiver-reported HRQoL in children with RDs.

Distress levels were remarkably high among the current sample of family caregivers of children with RDs. Clinical distress was reported by almost 90% of the caregivers. Notably, the mean distress score of the caregivers was well above the cut-off for clinical distress set by the authors of the original DT-P, highlighting the clinical relevance in this sample. Furthermore, caregivers experienced numerous problems in the domains of practical, physical, social/family, cognitive, emotional, and parenting issues, providing insights regarding the sources of distress. While all problem domain scores significantly correlated with overall distress, cognitive and practical problems showed the weakest and emotional problems the highest correlation, revealing possible targets for interventions. The majority of participants suffered from sleep problems, fatigue, being out of shape, weight problems, fears, feeling tense or nervous, worry, as well as problems regarding work/study, childcare/child supervision and the independence of their child, problems regarding leisure activities/relaxing, and keeping emotions under control.

The current findings are consistent with pre-pandemic studies demonstrating the immense burden and compromised psychosocial health of caregivers of children with chronic illnesses [46], and particularly in caregivers of children with RDs [12, 47, 48]. Given the negative impact of the COVID-19 pandemic on the mental health and well-being of children and their families in the general population [21, 22], it was reasonable to assume that the pandemic would particularly affect caregivers of children with RDs who were already highly strained before the pandemic. The assumption is further supported by findings of a significantly lower quality of life and significantly higher impairment in mental health in parents of children with a congenital RD compared to healthy controls and norm data in times of the early COVID-19 pandemic [28].

Family caregivers of children with RDs reported wide-ranging psychosocial information needs regarding the navigation through the health care system (e.g., financial aids related to the disease of the child), psychosocial strains in the family (e.g., coping with emotional strain), further support offerings (e.g., support options for everyday life), and the strengthening of oneself and the family system (e.g., strengthening of siblings). These findings suggest that the current provision of information by professionals or existing websites is perceived as inadequate by families and underscore the importance of additional support services. The current psychosocial information needs are consistent with previous studies that highlighted the need for more information about psychosocial support options and sociolegal counseling among family caregivers of children with RDs before the COVID-19 pandemic [6–8, 16–18, 49]. Since many of the pre-pandemic psychosocial counseling and care services were not available during the COVID-19 restrictions in Germany or were not used due to risk of infection, it can be assumed that psychosocial information needs increased even further during the course of the pandemic.

Most participants (136 of 140 participants, 97.1%) reported no helpful web pages devoted to psychosocial burdens, which is also consistent with the underrepresentation of psychosocial topics on specific RD information websites [18]. Other studies report that parents of children with RDs regularly use the internet as a source of information [17, 50] and that internet-sourced information was found to have a significant empowering effect on the parents [50]. These findings, along with the high demand for psychosocial information revealed by the current results, suggest that more informational websites on psychosocial matters are urgently needed. However, other possible explanations for this extremely high proportion should also be considered. It is possible that participants had difficulties assigning which topics belonged to “psychosocial burdens” and were therefore unable to name any specific websites. Moreover, caregivers may primarily search for specific topics via search engines and may not recall individual pages in their search history. Another possible explanation could be that caregivers tend to search more for practical information such as health and long-term care insurance benefits while neglecting their own psychosocial needs as they do not consider the internet to be useful for self-care-related purposes. Qualitative studies using interviews are warranted to investigate the role of online information in the care of families of children with RDs.

Almost half of the children with RDs over the age of four had an impaired HRQoL, as reported by their caregivers. The caregiver-reported HRQoL of the children with RDs was comparable to the parent-reported HRQoL of children with severe chronic diseases but substantially lower than that of children with mild to moderate chronic diseases [43]. However, it should be noted that the reference sample was not matched by age or gender, and no reference data for children under the age of 8 years exist for the measure. Because the age of the children in the current sample was not associated with caregiver-reported HRQoL scores, and norm differentiation is only recommended for background factors associated with the outcome variable, this can be considered appropriate [51]. Previous research indicates that the HRQoL of children with RDs was already impaired before the pandemic [52, 53] and that the quality of life of children generally decreased during the pandemic [21]. Therefore, it seems likely that the present finding is a combination of pre-pandemic impairment in HRQoL and additional impairment due to the pandemic. Note that the current data were collected at the onset of the pandemic and may not reflect its full impact on the HRQoL of children with RDs.

The distress level of the caregivers was positively associated with their overall psychosocial information need and negatively associated with the caregiver-reported HRQoL of the children with RDs, whereas psychosocial information needs and caregiver-reported HRQoL of the children were not found to be correlated. To our knowledge, no previous study has examined a direct relationship between distress levels and psychosocial information needs in family caregivers of children with RDs–although an existing relationship provides an interesting starting point for psychosocial support services in the form of information offers. The HRQoL of children with RDs appears to play an important role in caregiver burden, which is in line with previous research that has identified emotional and behavioral problems in children as important determinants of caregiver burden [12]. This also broadly fits with previous findings that the physical, emotional, social, and school functioning of children with RDs were associated with parental life satisfaction [52]. Altogether, the results presented also correspond well with previous research and theoretical frameworks on related constructs–for example, caregiver needs and models of stress, quality of life, or coping with stress [1, 9, 19, 54–58].

Research in times of the COVID-19 pandemic was primarily concerned with families of healthy children [21, 59], or children with disabilities in general [25], whereas little consideration was given so far to the psychosocial situation and needs of families with children with RDs [28]. However, the uncertainty about the impact of COVID-19 was even more threatening to people with chronic or pre-existing diseases and especially RDs [26, 27]. Furthermore, the German COVID-19 restrictions during the study period concerned particularly sensitive areas of life, including the closure of school and daycare, the cancellation of leisure activities, contact restrictions, restrictive visitation policies in medical facilities, and limited access to social support, which particularly affected individuals who needed special support and therapies. These pandemic-specific stressors, which added to pre-pandemic stressors, should be taken into account when interpreting the situation of the families.

The current results should be interpreted in light of the exploratory nature of the study and should be understood as a general inventory of the psychosocial situation of the families during the early COVID-19 pandemic. Even though the present research delivers interesting starting points for further support offerings, it is limited to providing a snapshot of the situation of family caregivers of children with RDs during the first year of the COVID-19 pandemic. Furthermore, the cross-sectional study design does not allow making inferences about causality, as the temporal link between the outcome and the exposure cannot be determined. More research with experimental or at least longitudinal study designs and setting up structural equation models or directed acyclic graphs [60] are needed to further investigate the nature of the discovered links. Moreover, embedding the presented results into theoretical frameworks on stress and needs in caregivers of children with disabilities (e.g., Perry’s stress framework for parents of children with disabilities [58]) would be desirable.

Analogous to the depression-distortion hypothesis [61, 62], the distress level of the caregivers may have biased their assessment of the HRQoL of their children [30, 31], which may have distorted the magnitude of the true association. However, cognitive biases are typical for people with confirmed depression, and a distinction should be made here from the present sample, in which no confirmed diagnoses but only elevated distress levels were found. Furthermore, not the psychopathology of the children, but their HRQoL was rated by the caregivers, and the “proxy problem” in HRQoL ratings may be smaller than previously assumed [63].

Some limitations regarding the representativeness of the sample restrict the generalizability of the results. First, the sample size is rather small considering the large population size in Germany. Nonetheless, smaller samples are relatively common in the field of RDs, and the current sample size is comparatively decent for a sample of family caregivers of children with RDs. Furthermore, it is plausible that mainly people who are involved in self-help or are otherwise proactive were reached. Notably, participants in the current study mostly included individuals with a high level of education. In addition, fathers caring for a child with RD were heavily underrepresented in the current sample, whose experiences and needs might differ from those of mothers–a problem that is quite prevalent in similar studies [64]. Future studies should include a more diverse field of participants–including children with RDs if possible–by acquiring through primary care providers or centers for RDs. In addition, the current results may not be generalizable to other countries with different health care or support systems. However, the consistency of the current results with those of previous studies from different countries suggests that the findings may be broadly applicable to other countries.

### Practical implications

The current study should raise awareness of the unmet psychosocial care needs of family caregivers of children with RDs and their heavily strained situation among practitioners and policymakers, particularly during the COVID-19 pandemic. Collectively, the presented results suggest that there is an acute need for action to ensure sufficient psychosocial care for family caregivers of children with RDs. Importantly, the COVID-19 pandemic should not compromise the psychosocial care for the families. More pre- and intervention options for the families are needed and psychosocial supports should be offered to all families in the diagnosis of an RD of their child, while health care professionals should be aware of the high prevalence of clinical distress among caregivers. Comprehensive care programs should be complemented by low-threshold offerings that can be integrated into the daily lives of those affected. As an example, the CARE-FAM-NET project, which is currently being evaluated, may take an important step towards improving access to psychosocial services in Germany and serve as a model for other countries [35].

Our findings underscore the importance of psychosocial information for caregivers. Psychosocial information and support options should become part of the standard medical care that is automatically provided when a child is diagnosed or suspected of having an RD. The existing guideline in Germany on psychosocial care in pediatric oncology [65] could serve as a basis for the development of a similar guideline for RDs. In addition, the wide reach of (umbrella) patient organizations for RDs (e.g., ACHSE for Germany, NORD for the USA) should be leveraged to disseminate existing information services among families with children with RDs. A feasible avenue is to provide a modular website–such as the CARE-FAM-NET website [66], which we built for families with children with RDs using the current findings on psychosocial information needs. Any such website can cover different topics that are of relevance for the families and can be accessed individually depending on acute need. Furthermore, existing websites that provide medical information to families with children with RDs should be used to also provide psychosocial information, for example, by linking to quality-checked websites with psychosocial information. Finally, psychosocial care for family caregivers of children with RDs should be given greater focus within existing international collaboration projects, as multinational programs can have a great impact and significantly advance the dissemination of scientific knowledge. The existing European Reference Networks for RDs (ERNs) could, for example, develop a guide for health care professionals on the psychosocial needs of families affected by RDs alongside better access to psychosocial care to promote resilience.

## Conclusions

The psychosocial situation of families living with children with RDs during the early COVID-19 pandemic was characterized by high caregiver distress, unmet psychosocial information needs, and impaired HRQoL in their children with RDs. Health care professionals need to be alert to the high distress among caregivers of children with RDs throughout and after the COVID-19 pandemic. For family-centered psychosocial support services to be integrated into standard care, future studies are warranted to investigate the possible effect of providing psychosocial support and information on distress levels in caregivers of children with RDs.

## Supplementary Information

**Additional file 1**. Qualitative pre-analysis

**Additional file 2**. Original German version of the psychosocial information need questionnaire

**Additional file 3**. Psychosocial information needs of family caregivers of a child with RD

## Data Availability

The dataset analyzed during the current study is available from the corresponding author on reasonable request.

## Abbreviations

RD: Rare disease
COVID-19: Coronavirus disease 19
HRQoL: Health-related quality of Life
DT-P: Distress thermometer for parents
DCGM: DISABKIDS chronic generic measure
CARE-FAM-NET: Children affected by rare diseases and their families-network

## References

[1] Lee CKJ. So rare, who cares? A study of stress and coping of parents of children with rare diseases in Hong Kong [Dissertation]. Hong Kong: The Hong Kong Polytechnic University; 2018.

[2] Luzzatto L, Hollak CEM, Cox TM, Schieppati A, Licht C, Kääriäinen H (2015). Rare diseases and effective treatments: are we delivering?. Lancet.

[3] Cardinali P, Migliorini L, Rania N (2019). The caregiving experiences of fathers and mothers of children with rare diseases in Italy: challenges and social support perceptions. Front Psychol.

[4] Domaradzki J, Walkowiak D (2019). Medical students’ knowledge and opinions about rare diseases: a case study from Poland. Intractable Rare Dis Res.

[5] Julkowska D, Austin CP, Cutillo CM, Gancberg D, Hager C, Halftermeyer J (2017). The importance of international collaboration for rare diseases research: a European perspective. Gene Ther.

[6] Pelentsov LJ, Laws TA, Esterman AJ (2015). The supportive care needs of parents caring for a child with a rare disease: a scoping review. Disabil Health J.

[7] Pelentsov LJ, Fielder AL, Laws TA, Esterman AJ (2016). The supportive care needs of parents with a child with a rare disease: results of an online survey. BMC Fam Pract.

[8] Anderson M, Elliott EJ, Zurynski YA (2013). Australian families living with rare disease: experiences of diagnosis, health services use and needs for psychosocial support. Orphanet J Rare Dis.

[9] Baumbusch J, Mayer S, Sloan-Yip I (2018). Alone in a crowd? Parents of children with rare diseases’ experiences of navigating the healthcare system. J Genet Couns.

[10] Brewer HM, Eatough V, Smith JA, Stanley CA, Glendinning NW, Quarrell OW (2008). The impact of Juvenile Huntington’s disease on the family: the case of a rare childhood condition. J Health Psychol.

[11] Pelentsov LJ, Fielder AL, Esterman AJ (2016). The supportive care needs of parents with a child with a rare disease: a qualitative descriptive study. J Pediatr Nurs.

[12] Fitzgerald J, Gallagher L (2021). Parental stress and adjustment in the context of rare genetic syndromes: a scoping review. J Intellect Disabil.

[13] Morgenstern L, Wagner M, Denecke J, Grolle B, Johannsen J, Wegscheider K (2017). The need for psychosocial support in parents of chronically ill children/ Psychosozialer Unterstutzungsbedarf von Eltern mit schwer chronisch somatisch erkrankten Kindern. Prax Kinderpsychol Kinderpsychiatr.

[14] Douma M, Bouman CP, van Oers HA, Maurice-Stam H, Haverman L, Grootenhuis MA (2020). Matching psychosocial support needs of parents of a child with a chronic illness to a feasible intervention. Matern Child Health J.

[15] McMullan J, Crowe AL, Bailie C, Moore K, McMullan LS, Shamandi N (2020). Improvements needed to support people living and working with a rare disease in Northern Ireland: current rare disease support perceived as inadequate. Orphanet J Rare Dis.

[16] Litzkendorf S, Babac A, Rosenfeldt D, Schauer F, Hartz T, Lührs V (2016). Information needs of people with rare diseases-what information do patients and their relatives require. J Rare Diagn Ther.

[17] Crowe A, McKnight AJ, McAneney H (2019). Communication needs for individuals with rare diseases within and around the healthcare system of Northern Ireland. Front Public Health.

[18] Pauer F, Litzkendorf S, Göbel J, Storf H, Zeidler J, von der Schulenburg JM (2017). Rare diseases on the internet: an assessment of the quality of online information. J Med Internet Res.

[19] Resch JA, Benz MR, Elliott TR (2012). Evaluating a dynamic process model of wellbeing for parents of children with disabilities: a multi-method analysis. Rehabil Psychol.

[20] Fordham L, Gibson F, Bowes J (2012). Information and professional support: Key factors in the provision of family-centred early childhood intervention services. Child Care Health Dev.

[21] Ravens-Sieberer U, Kaman A, Erhart M, Otto C, Devine J, Löffler C (2021). Quality of life and mental health in children and adolescents during the first year of the COVID-19 pandemic: results of a two-wave nationwide population-based study. Eur Child Adolesc Psychiatry.

[22] Calvano C, Engelke L, Di Bella J, Kindermann J, Renneberg B, Winter SM (2021). Families in the COVID-19 pandemic: parental stress, parent mental health and the occurrence of adverse childhood experiences—results of a representative survey in Germany. Eur Child Adolesc Psychiatry.

[23] Wauters A, Vervoort T, Dhondt K, Soenens B, Vansteenkiste M, Morbée S (2022). Mental health outcomes among parents of children with a chronic disease during the COVID-19 pandemic: the role of parental burn-out. J Pediatr Psychol.

[24] Aishworiya R, Kang YQ (2021). Including children with developmental disabilities in the equation during this COVID-19 pandemic. J Autism Dev Disord.

[25] Masi A, Mendoza Diaz A, Tully L, Azim SI, Woolfenden S, Efron D (2021). Impact of the COVID-19 pandemic on the well-being of children with neurodevelopmental disabilities and their parents. J Paediatr Child Health.

[26] Halley MC, Stanley T, Maturi J, Goldenberg AJ, Bernstein JA, Wheeler MT (2021). “It seems like COVID-19 now is the only disease present on Earth”: living with a rare or undiagnosed disease during the COVID-19 pandemic. Genet Med.

[27] Hacker KA, Briss PA, Richardson L, Wright J, Petersen R (2021). COVID-19 and chronic disease: the impact now and in the future. Prev Chronic Dis.

[28] Fuerboeter M, Boettcher J, Barkmann C, Zapf H, Nazarian R, Wiegand-Grefe S (2021). Quality of life and mental health of children with rare congenital surgical diseases and their parents during the COVID-19 pandemic. Orphanet J Rare Dis.

[29] The Rare Reality of Covid-19. Genetic Alliance UK; 2020. Accessed 11 May 2022. https://geneticalliance.org.uk/gauk-news/news/the-rare-reality-of-covid-19-july-2020/.

[30] Diederen K, Haverman L, Grootenhuis MA, Benninga MA, Kindermann A (2018). Parental distress and quality of life in pediatric inflammatory bowel disease: implications for the outpatient clinic. J Pediatr Gastroenterol Nutr.

[31] Bramuzzo M, De Carlo C, Arrigo S, Pavanello PM, Canaletti C, Giudici F (2020). Parental psychological factors and quality of life of children with inflammatory bowel disease. J Pediatr Gastroenterol Nutr.

[32] Dunst CJ, Trivette CM (2009). Meta-analytic structural equation modeling of the influences of family-centered care on parent and child psychological health. Int J Pediatr.

[33] Grootenhuis MA, Bronner MB (2009). Paediatric illness! Family matters. Acta Paediatr.

[34] Barlow JH, Ellard DR (2006). The psychosocial well-being of children with chronic disease, their parents and siblings: an overview of the research evidence base. Child Care Health Dev.

[35] Boettcher J, Filter B, Denecke J, Hot A, Daubmann A, Zapf A (2020). Evaluation of two family-based intervention programs for children affected by rare disease and their families – research network (CARE-FAM-NET): study protocol for a rater-blinded, randomized, controlled, multicenter trial in a 2x2 factorial design. BMC Fam Pract.

[36] Schilling J, Tolksdorf K, Marquis A, Faber M, Pfoch T, Buda S (2021). Die verschiedenen Phasen der COVID-19-Pandemie in Deutschland: Eine deskriptive Analyse von Januar 2020 bis Februar 2021. Bundesgesundheitsblatt - Gesundheitsforschung - Gesundheitsschutz.

[37] Haverman L, van Oers HA, Limperg PF, Houtzager BA, Huisman J, Darlington AS (2013). Development and validation of the distress thermometer for parents of a chronically ill child. J Pediatr.

[38] van Oers HA, Schepers SA, Grootenhuis MA, Haverman L (2017). Dutch normative data and psychometric properties for the distress thermometer for parents. Qual Life Res.

[39] Roth AJ, Kornblith AB, Batel-Copel L, Peabody E, Scher HI, Holland JC (1998). Rapid screening for psychologic distress in men with prostate carcinoma. Cancer.

[40] Donovan KA, Grassi L, McGinty HL, Jacobsen PB (2014). Validation of the distress thermometer worldwide: state of the science. Psychooncology.

[41] Basart H, van Oers HA, Paes EC, Breugem CC, Don Griot JPW, van der Horst CM (2017). Health-related quality of life in children with Robin sequence. Am J Med Genet A.

[42] Bai X, Wang A, Cross W, Lam L, Plummer V, Guan Z (2020). Validation of the distress thermometer for caregivers of children and adolescents with schizophrenia. J Adv Nurs.

[43] The DISABKIDS Group Europe (2006). The DISABKIDS questionnaires: quality of life questionnaires for children with chronic conditions - Handbook.

[44] Muehlan H. Developing the DCGM-12: A short-form of the DISABKIDS condition-generic module assessing health related quality of life in children and adolescents with chronic conditions [Dissertation]. Hamburg: Staats-und Universitätsbibliothek Hamburg Carl von Ossietzky; 2010.

[45] Carona C, Silva N, Moreira H, Canavarro MC, Bullinger M (2015). Does the small fit them all? The utility of Disabkids-10 Index for the assessment of pediatric health-related quality of life across age-groups, genders, and informants. J Child Health Care.

[46] Cohn LN, Pechlivanoglou P, Lee Y, Mahant S, Orkin J, Marson A (2020). Health outcomes of parents of children with chronic illness: a systematic review and meta-analysis. J Pediatr.

[47] Picci RL, Oliva F, Trivelli F, Carezana C, Zuffranieri M, Ostacoli L (2015). Emotional burden and coping strategies of parents of children with rare diseases. J Child Fam Stud.

[48] Mori Y, Downs J, Wong K, Heyworth J, Leonard H (2018). Comparing parental well-being and its determinants across three different genetic disorders causing intellectual disability. J Autism Dev Disord.

[49] Currie G, Szabo J (2019). “It is like a jungle gym, and everything is under construction”: the parent’s perspective of caring for a child with a rare disease. Child Care Health Dev.

[50] Nicholl H, Tracey C, Begley T, King C, Lynch AM (2017). Internet use by parents of children with rare conditions: findings from a study on parents’ web information needs. J Med Internet Res.

[51] Goldhammer F, Hartig J, Moosbrugger H, Kelava A (2012). Interpretation von Testresultaten und Testeichung. Testtheorie und Fragebogenkonstruktion.

[52] Johansen H, Dammann B, Andresen IL, Fagerland MW (2013). Health-related quality of life for children with rare diagnoses, their parents’ satisfaction with life and the association between the two. Health Qual Life Outcomes.

[53] Witt S, Kolb B, Bloemeke J, Mohnike K, Bullinger M, Quitmann J (2019). Quality of life of children with achondroplasia and their parents—a German cross-sectional study. Orphanet J Rare Dis.

[54] Northouse LL, Katapodi MC, Schafenacker AM, Weiss D (2012). The impact of caregiving on the psychological well-being of family caregivers and cancer patients. Semin Oncol Nurs.

[55] Davies S, Hall D (2005). “Contact a family”: professionals and parents in partnership. Arch Dis Child.

[56] King G, King S, Rosenbaum P, Goffin R (1999). Family-centered caregiving and well-being of parents of children with disabilities: Linking process with outcome. J Pediatr Psychol.

[57] Nehring I, Riedel C, Baghi L, Moshammer-Karb T, Schmid R, Kries RV (2015). Psychosoziale Lage von Familien mit chronisch kranken Kindern: Eine Befragung betroffener Eltern in Selbsthilfegruppen. Gesundheitswesen.

[58] Perry A (2004). A model of stress in families of children with developmental disabilities: clinical and research applications. J Dev Disabil.

[59] Huebener M, Waights S, Spiess CK, Siegel NA, Wagner GG (2021). Parental well-being in times of covid-19 in Germany. Rev Econ Househ.

[60] Rohrer J (2018). Thinking clearly about correlations and causation: graphical causal models for observational data. Adv Methods Pract Psychol Sci.

[61] Richters JE (1992). Depressed mothers as informants about their children: a critical review of the evidence for distortion. Psychol Bull.

[62] Müller JM, Achtergarde S, Furniss T (2011). The influence of maternal psychopathology on ratings of child psychiatric symptoms: an SEM analysis on cross-informant agreement. Eur Child Adolesc Psychiatry.

[63] Sattoe JN, van Staa A, Moll HA, On Your Own Feet Research Group (2012). The proxy problem anatomized: child-parent disagreement in health related quality of life reports of chronically ill adolescents. Health Qual Life Outcomes.

[64] Boettcher J, Boettcher M, Wiegand-Grefe S, Zapf H (2021). Being the pillar for children with rare diseases-a systematic review on parental quality of life. Int J Environ Res Public Health.

[65] Schröder HM, Lilienthal S, Schreiber-Gollwitzer BM, Grießmeier B, Hesselbarth B, Lein-Köhler I, et al. S3-Guideline “Psychosocial Care in Paediatric Oncology and Haematology”. https://register.awmf.org/de/leitlinien/detail/025-002. Accessed 16 Aug 2022.

[66] Dreier M, Rezvani F, Rihm L, Busch N, Glowacz A, Dirmaier J. CARE-FAM-NET | Für Kinder mit seltenen Erkrankungen, deren Eltern und Geschwister. 2021. Accessed 12 Oct 2021. https://www.carefamnet.org/fuer-familien/.

