# Supplementary material for: The psychosocial situation of families caring for children with rare diseases during the COVID-19 pandemic: results of a cross-sectional online survey
Source: Orphanet J Rare Dis. 2022 Dec 26;17:449. doi: 10.1186/s13023-022-02595-0 (PMC9791975; doi:10.1186/s13023-022-02595-0)
Supplement: Supplementary file 1 — Additional file 1. Qualitative pre-analysis [file 13023_2022_2595_MOESM1_ESM.docx]

# Additional file 1. Qualitative pre-analysis

In order to identify the psychosocial information needs of family caregivers of children with rare diseases (RDs) that should be covered within the quantitative online survey, semi-structured telephone interviews with 10 family caregivers of children with RDs and eight experts in the field were conducted in the third calendar quarter of 2019. Interviewers were student- and research assistants from the Institute of Medical Psychology (Medical Center Hamburg-Eppendorf). The interview guideline included: 1. Demographics (caregivers)/ occupation-related information (experts), 2. RD of the child (caregivers), 3. Psychosocial strains due to the RD, as experienced by caregivers or assessed by experts, 4. Need for an online psychosocial support offering for affected families, 5. Relevance of specific psychosocial contents that might be covered on a website (derived from theoretical considerations, prior research, and input from experts and family caregivers, rating 1–10). Thematic content analyses of the interview transcripts using MAXQDA included deductive assignments of statements to the pre-defined information categories as well as inductive coding of new content. Caregivers consisted of eight mothers, one father, and one sister of collectively 12 children (1x3 and 9x1) with a confirmed RD. The mean age of caregivers was 44.6 years (*SD* = 8.36), the mean age of their children with RD was 16.3 years (*SD* = 7.98). In seven of the 10 families, there were healthy siblings (1x1 and 6x2). Time since diagnosis was on average 9.20 years (*SD* = 6.56), range 0.5–20 years. The experts came from the field of self-help (*n* = 5) or were involved in the psychosocial care of affected families as psychotherapist/ head of the medical psychology department (each *n* = 1) and pediatric endocrinologist (*n* = 1). The participants named various psychosocial strains related to caring for a child with RD (e.g., additional organizational work, lack of information by HCPs, social exclusion of the child, legal disputes) and adverse adaptions (e.g., worry about neglecting healthy siblings, existential fears, feelings of powerlessness, grief, guilt, sleep disturbances, difficulty acting in a self-caring manner). Importance ratings (*Median*, *IQR*) regarding the pre-defined information categories were moderate to high: Comprehending the RD and explaining it to others, 9.5 (7.75–10); navigating the health-care system, 9.5 (6.5–10); emotional concerns related to the RD, 8.5 (7.25–9.25); (financial) costs related to the RD, 8 (7–10). Some expressed that the information was important but very individualized and therefore difficult to implement on a website. Participants rated available offerings as expandable and named further information needs, e.g., on (emotional) coping with the diagnosis, social-legal issues, available care offerings (medical, psychosocial, self-help), practical advice. Results informed the development of the questionnaire on psychosocial information needs.
