# Supplementary material for: The psychosocial situation of families caring for children with rare diseases during the COVID-19 pandemic: results of a cross-sectional online survey
Source: Orphanet J Rare Dis. 2022 Dec 26;17:449. doi: 10.1186/s13023-022-02595-0 (PMC9791975; doi:10.1186/s13023-022-02595-0)
Supplement: Supplementary file 2 — Additional file 2. Original German version of the psychosocial information need questionnaire [file 13023_2022_2595_MOESM2_ESM.docx]

# Additional file 2. Original German version of the psychosocial information need questionnaire

| **Psychosoziale Informationsbedarfe für Eltern im Zusammenhang mit der Erkrankung des Kindes**  Wir möchten auf einer Webseite Informationen für Familien mit Kindern mit Seltenen Erkrankungen zur Verfügung stellen. Dabei soll es vor allem um seelische und soziale Belastungen und Anforderungen gehen, die im Zusammenhang mit der Seltenen Erkrankung eines Kindes entstehen können. Medizinische Informationen, z.B. zur Erkrankung oder Behandlungsmöglichkeiten, stehen dabei nicht im Fokus.  Wir bitten um eine Einschätzung zu den Bereichen:   - Zurechtfinden im Gesundheitssystem - Seelische/emotionale Belastungen in der Familie - Sich stärken, um für andere stark zu sein - Weitere Unterstützungsangebote.   Um die noch zu entwickelnden Inhalte gut auf Ihre Bedarfe abzustimmen, brauchen wir Ihre Hilfe. Bitte schätzen Sie dafür im Folgenden auf einer Skala zwischen 1 und 5 ein, ob oder wie ausführlich auf solch einer Webseite folgende Informationen angeboten werden sollten. „1“ bedeutet dabei, dass Sie keine Informationen benötigen, „5“ bedeutet, dass Sie ausführliche Informationen zum entsprechenden Bereich benötigen: |
| --- |
| Zurechtfinden im Gesundheitssystem: Bitte schätzen Sie Ihren Informationsbedarf für die Webseite ein.  Bitte wählen Sie die zutreffende Antwort für jeden Punkt aus:   \| 1 Keine  Informationen benötigt…  ⃝ \| 2  ⃝ \| 3  ⃝ \| 4  ⃝ \| 5 Ausführliche Informationen benötigt…  ⃝ \| \| --- \| --- \| --- \| --- \| --- \| |
| ... darüber, wie die deutsche Gesundheitsversorgung strukturiert ist. |
| … darüber, wo man spezifische Informationen über die Erkrankung des Kindes und/oder Behandlungsmöglichkeiten erhält. |
| …über Leistungen im Rahmen des Teilhabegesetzes („Teilhabe und Inklusion: Was für Rechte und Möglichkeiten habe ich?“). |
| … zur Feststellung des Grads der Behinderung (GdB) und Beantragung eines Schwerbehindertenausweises. |
| … darüber, wie meine Familie und/oder ich psychologische Beratungsangebote finden und in Anspruch nehmen kann. |
| … über finanzielle Unterstützungsmöglichkeiten im Zusammenhang mit der Erkrankung des Kindes (z.B. Zuzahlungen bei Behandlungsmaßnahmen, notwendige Unterstützung im Alltag). |
| … darüber, welche Leistungen wann von der Kranken- oder Pflegeversicherung übernommen werden. |
| … über aktuelle Gesetzesänderungen (z.B. Änderungen des Teilhabegesetzes oder der Pflegereform). |
| … über spezielle Rehabilitationsmaßnahmen (z.B. Eltern-Kind-Kur, Kurangebote speziell für Kinder mit Behinderungen). |
| … über Palliativ- und Hospizmedizin. |
| … in Form einer Zusammenstellung von weiteren Hilfsangeboten (neben Leistungen der gesetzlichen Krankenkassen). |
| … darüber, wie ich am besten mit Ärzt*innen und medizinischem Fachpersonal sprechen kann. |
| … darüber, wie bzw. wo ich spezialisierte Kliniken, Zentren und/oder Expert*innen für seltene Erkrankungen finden kann. |
| … über den Übergang des Kindes in die medizinische Versorgung für Erwachsene. |
| … über den Schulaustritt des Kindes („Was für Möglichkeiten hat mein Kind nach der Schule?“). |
| … über juristische Probleme (z.B. Sozialrecht, nützliche Urteile, Widerspruch). |
| … über Hilfsmöglichkeiten ohne Diagnose (z.B. Anlaufstellen für Kinder mit bisher nicht diagnostizierten Erkrankungen). |
| (continued) |
|  |
| (continued) |
| Seelische/emotionale Belastungen in der Familie: Bitte schätzen Sie Ihren Informationsbedarf für die Webseite ein.  Bitte wählen Sie die zutreffende Antwort für jeden Punkt aus:   \| 1 Keine  Informationen benötigt…  ⃝ \| 2  ⃝ \| 3  ⃝ \| 4  ⃝ \| 5 Ausführliche Informationen benötigt…  ⃝ \| \| --- \| --- \| --- \| --- \| --- \| |
| … darüber, welche emotionalen Belastungen (z.B. Ängste, Trauer, Wut, Einsamkeit) im Allgemeinen in Zusammenhang mit der Erkrankung eines Kindes auftreten können. |
| … darüber, wie ich mit emotionalen Belastungen (z.B. Ängste, Trauer, Wut, Einsamkeit) im Zusammenhang mit der Erkrankung des Kindes umgehen kann. |
| … darüber, wie meine Familie mit emotionalen Belastungen (z.B. Ängste, Trauer, Wut, Einsamkeit) aufgrund der Erkrankung des Kindes umgehen kann. |
| … über mögliche emotionale Belastungen der Geschwisterkinder aufgrund der Erkrankung des Kindes (z.B. Ängste, Trauer, Wut, Einsamkeit). |
| … darüber, wie ich meine momentane psychische Belastung einschätzen kann (z.B. über Selbsttests, die auf der Webseite durchgeführt werden können). |
| … darüber, wie ich selbst die Erkrankung des Kindes und deren Folgen besser verstehen kann. |
| … darüber, wie ich die Erkrankung des Kindes und deren Folgen anderen (z.B. Freunden, Arbeitskollegen) besser erklären kann. |
| … darüber, wie ich mit Partnerschaftsproblemen besser umgehen kann. |
| … in Form von Erfahrungsberichten von anderen Betroffenen (z.B. „Was hat meiner Familie geholfen?“; „Wie sind wir mit Belastungen umgegangen?“; „Welche Angebote haben uns geholfen?“). |
| … darüber, wie ich das Kind bei seiner sozialen Einbindung unterstützen kann. |
| … darüber, wie ich mit meinen Gefühlen nach der Diagnosestellung umgehen kann. |
| Sich stärken, um für andere stark zu sein: Bitte schätzen Sie Ihren Informationsbedarf für die Webseite ein.  Bitte wählen Sie die zutreffende Antwort für jeden Punkt aus:   \| 1 Keine  Informationen benötigt…  ⃝ \| 2  ⃝ \| 3  ⃝ \| 4  ⃝ \| 5 Ausführliche Informationen benötigt…  ⃝ \| \| --- \| --- \| --- \| --- \| --- \| |
| … darüber, wie ich mein Gesundheitsverhalten verbessern kann (z.B. weniger Alkohol, mehr körperliche Aktivität, besserer Schlaf). |
| … darüber, wie ich besser entspannen kann (z.B. mit Hilfe von Entspannungstechniken). |
| … darüber, wie ich mehr Ausgleich in der Freizeit finde. |
| … darüber, wie ich mehr am sozialen Leben teilhaben kann. |
| … darüber, wie ich seelischen Erkrankungen vorbeugen kann (z.B. Depression). |
| … darüber, wie ich meine Partnerschaft stärken kann. |
| … darüber, wie ich Geschwisterkinder stärken kann. |
| … darüber, wie ich Beruf und Pflege des Kindes vereinbaren kann. |
| Weitere Unterstützungsangebote: Bitte schätzen Sie Ihren Informationsbedarf für die Webseite ein.  Bitte wählen Sie die zutreffende Antwort für jeden Punkt aus:   \| 1 Keine  Informationen benötigt…  ⃝ \| 2  ⃝ \| 3  ⃝ \| 4  ⃝ \| 5 Ausführliche Informationen benötigt…  ⃝ \| \| --- \| --- \| --- \| --- \| --- \| |
| … über Selbsthilfeangebote (z.B. Selbsthilfegruppen oder Foren). |
| … über Unterstützungsmöglichkeiten im Alltag. |
| … darüber, welche regelmäßigen Betreuungsmöglichkeiten es für das Kind gibt. |
| … darüber, welche Möglichkeiten zur Freizeitgestaltung/Hobbies es für das Kind gibt. |
| … über Ferienbetreuung des Kindes. |
| … über Ansprechpartner vor Ort (z.B. Behindertenbeauftragte der Stadt). |
| ... über Möglichkeiten zum Erfahrungsaustausch (z.B. Online-Foren). |
