# Supplementary material for: The psychosocial situation of families caring for children with rare diseases during the COVID-19 pandemic: results of a cross-sectional online survey
Source: Orphanet J Rare Dis. 2022 Dec 26;17:449. doi: 10.1186/s13023-022-02595-0 (PMC9791975; doi:10.1186/s13023-022-02595-0)
Supplement: Supplementary file 3 — Additional file 3. Psychosocial information needs of family caregivers of a child with RD [file 13023_2022_2595_MOESM3_ESM.docx]

# Additional file 3. Psychosocial information needs of family caregivers of children with RDs

| **Navigating the health care system** | ***M (SD)*** | ***n (*%*) 4/5* ^a^** | ***Mode*** | ***Mdn (IQR)*** | |
| --- | --- | --- | --- | --- | --- |
| Information about how the German health care system is structured. | 3.54 (1.28) | 79 (53.0) | 5 | 4 (3–5) | |
| Information on where to find specific information about the disease of the child and/or treatment options. | 4.28 (1.17) | 121 (81.2) | 5 | 5 (4–5) | |
| Information about benefits within the framework of the “federal participation act” [Bundesteilhabegesetz] (“participation and inclusion: What are my rights and options?”). | 4.48 (1.00) | 125 (83.9) | 5 | 5 (4–5) | |
| Information about determining/assessing the level of disability [Grad der Behinderung] and applying for a severely disabled pass [Schwerbehindertenausweis]. | 3.97 (1.38) | 106 (71.1) | 5 | 5 (3–5) | |
| Information about how my family and/or I can find and access psychological counseling. | 4.20 (1.08) | 113 (75.8) | 5 | 5 (4–5) | |
| Information about financial aid related to the disease of the child (e.g., co-payments for treatments, necessary support in everyday life). | 4.34 (1.03) | 122 (81.9) | 5 | 5 (4–5) | |
| Information about services covered by health- and/or nursing care insurance. | 4.39 (0.95) | 124 (83.2) | 5 | 5 (4–5) | |
| Information about recent law changes (e.g., change of the participation act or the care reform). | 4.21 (1.11) | 112 (75.2) | 5 | 5 (4–5) | |
| Information about special rehabilitation measures (e.g., parent-child measures, rehabilitation measures specifically for children with disabilities). | 4.49 (0.91) | 131 (87.9) | 5 | 5 (4–5) | |
| Information about palliative and hospice medicine. | 2.93 (1.51) | 55 (36.9) | 1 | 3 (1–4) | |
| Information as a collection of further support options (in addition to benefits covered by statutory health insurances). | 4.32 (0.92) | 126 (84.6) | 5 | 5 (4–5) | |
| Information about how to communicate with doctors and healthcare professionals. | 3.60 (1.30) | 92 (61.7) | 4;5 | 4 (3–5) | |
| Information about how and where I can find specialized clinics, centers, and/or experts for rare diseases. | 4.14 (1.20) | 118 (79.2) | 5 | 5 (4–5) | |
| Information about the child’s transition to adult medical care. | 4.05 (1.30)  4.34 (1.18) ^b^ | 109 (73.2)  50 (86.2) ^b^ | 5  5 ^b^ | 5 (3–5)  5 (4–5) ^b^ | |
| Information about the child leaving school ("What options does my child have after school?"). | 3.87 (1.37)  4.19 (1.25) ^b^ | 97 (65.1)  45 (77.6) ^b^ | 5  5 ^b^ | 4 (3–5)  5 (4–5) ^b^ | |
| Information about legal issues (e.g., social law, useful judgments, objections). | 4.12 (1.11) | 110 (73.8) | 5 | 5 (3–5) | |
| Information about support options without a confirmed diagnosis (e.g., contact points for children with unclear diagnoses). | 3.23 (1.61)  4.77 (0.60) ^c^ | 73 (49.0)  12 (92.3) ^c^ | 5  5 ^c^ | 3 (1–5)  5 (5–5) ^c^ | |
| **Psychosocial (i.e., mental, emotional, social) strain in the family** | ***M (SD)*** | ***n (*%*) 4/5* ^a^** | ***Mode*** | ***Mdn (IQR)*** | |
| Information about emotional strains (e.g., fear, sadness, anger, loneliness) that may arise in connection with a child’s disease. | 3.91 (1.08) | 100 (67.1) | 5 | 4 (3–5) | |
| Information about how I can cope with emotional strains (e.g., fear, sadness, anger, loneliness) in connection with the disease of the child. | 4.11 (1.04) | 114 (76.5) | 5 | 4 (4–5) | |
| Information about how my family can cope with emotional strain (e.g., fear, sadness, anger, loneliness) in connection with the disease of the child. | 4.09 (1.08) | 110 (73.8) | 5 | 4 (3–5) | |
| Information about possible emotional reactions of siblings due to the disease of the child (e.g., fear, sadness, anger, loneliness). | 3.90 (1.44)  4.45 (0.94) ^d^ | 106 (71.1)  77 (86.5) ^d^ | 5  5 ^d^ | 5 (3–5)  5 (4–5) ^d^ | |
| Information about how I can assess my current psychological strain (e.g., via self-tests that can be carried out on the website). | 3.83 (1.25) | 92 (61.7) | 5 | 4 (3–5) | |
| Information about how I myself can better understand the disease of the child and its consequences. | 3.84 (1.19) | 102 (68.5) | 5 | 4 (3–5) | |
| Information about how I can better explain the disease of the child and its consequences to others (e.g., friends, work colleagues). | 3.70 (1.26) | 88 (59.1) | 5 | 4 (3–5) | |
| Information about how I can better deal with relationship problems. | 3.33 (1.44)  3.29 (1.43) ^e^ | 72 (48.3)  51 (46.8) ^e^ | 5  5 ^e^ | 3 (2–5)  3 (2–5) ^e^ | |
| Information through experience reports from other people affected (e.g., "What has helped my family?"; "How did we deal with the burden?"; "Which offerings have helped us?"). | 3.95 (1.16) | 106 (71.1) | 5 | 4 (3–5) | |
| Information about how to support the social inclusion of the child. | 4.07 (1.08) | 106 (71.1) | 5 | 4 (3–5) | |
| Information about how to deal with my feelings after the diagnosis. | 3.64 (1.37)  4.54 (0.88) ^f^ | 88 (59.1)  10 (76.9) ^f^ | 5  5 ^f^ | 4 (3–5)  5 (4–5) ^f^ | |
| **Strengthen yourself to be strong for others** | ***M (SD)*** | ***n (*%*) 4/5* ^a^** | ***Mode*** | ***Mdn (IQR)*** | |
| Information about how I can improve my health behavior (e.g., less alcohol, more physical activity, better sleep). | 2.93 (1.37) | 51 (34.2) | 3 | 3 (2–4) | |
| Information about how I can relax better (e.g., with the help of relaxation techniques). | 3.30 (1.26) | 70 (47.0) | 4 | 3 (2–4) | |
| Information about how I can find better balance in leisure time. | 3.17 (1.29) | 58 (38.9) | 3 | 3 (2–4) | |
| Information about how I can participate more in social life. | 3.03 (1.36) | 56 (37.6) | 3 | 3 (2–4) | |
| Information about how I can prevent mental illness. | 3.42 (1.31) | 76 (51.0) | 5 | 4 (2–5) | |
| Information about how I can strengthen my partnership. | 3.12 (1.40)  3.10 (1.37) ^e^ | 65 (43.6)  48 (44.0) ^e^ | 4  4 ^e^ | 3 (2–4)  3 (2–4) ^e^ | |
| Information about how I can strengthen siblings. | 3.67 (1.50)  4.25 (1.00) ^d^ | 100 (67.1)  77 (86.5) ^d^ | 5  5 ^d^ | 4 (3–5)  4 (4–5) ^d^ | |
| Information about how I can combine work and caring for the child. | 3.57 (1.48) | 87 (58.4) | 5 | 4 (2.5–5) | |
| **Further support offerings** | ***M (SD)*** | ***n (*%*) 4/5* ^a^** | ***Mode*** | ***Mdn (IQR)*** | |
| Information about self-help offerings (e.g., support groups or forums). | 3.77 (1.31) | 91 (61.1) | 5 | 4 (3–5) | |
| Information about support options in everyday life. | 4.07 (1.16) | 110 (73.8) | 5 | 4 (3–5) | |
| Information about regular care options for the child. | 3.83 (1.33) | 96 (64.4) | 5 | 4 (3–5) | |
| Information about the possibilities for leisure activities/hobbies for the child. | 3.74 (1.28) | 90 (60.4) | 5 | 4 (3–5) | |
| Information about holiday care options for the child. | 3.50 (1.46) | 82 (55.0) | 5 | 4 (2–5) | |
| Information about local points of contact. | 3.85 (1.32) | 96 (64.4) | 5 | 4 (3–5) | |
| Information about opportunities to share experiences (e.g., online forums). | 3.58 (1.33) | 84 (56.4) | 5 | 4 (3–5) | |
| *Note.* RD: rare disease. *N* = 149. The response format was a five-point Likert scale with options ranging from 1 (*no information needed*) to 5 (*in-depth information needed*). The median and interquartile ranges are reported in addition to average ratings, due to non-normality of ratings. Further, the mode is reported, as it is of interest, what most people rated. Some item scores are additionally reported for sub-samples, as the content assumedly only impacts these defined subgroups.  ^a^ number (percentage) of ‘4’ or ‘5’ ratings for the respective item. ^b^ only caregivers whose children are between 10–20 years old, *N* = 58. ^c^ only caregivers with children without a secured diagnosis, *N* = 13. ^d^ only caregivers with healthy siblings in the household, *N* = 89. ^e^ only parents in a partnership, *N* = 109. ^f^ only when time since diagnosis is < 1 year, *N* = 13. | | | | |  |
